# Role of lamp type in conventional batch and micro-photoreactor for photocatalytic hydrogen production

**DOI:** 10.3389/fchem.2023.1271410

**Published:** 2023-09-20

**Authors:** Vendula Meinhardová, Lada Dubnová, Helena Drobná, Lenka Matějová, Kamila Kočí, Libor Čapek

**Affiliations:** ^1^ Faculty of Chemical Technology, University of Pardubice, Pardubice, Czechia; ^2^ Institute of Environmental Technology, VŠB-Technical University of Ostrava, Ostrava Poruba, Czechia

**Keywords:** irradiation, intensity, UV-LED lamp, hydrogen production, micro-photoreactor, batch reactor

## Abstract

The use of an irradiation source with a homogeneous distribution of irradiation in the volume of the reaction mixture belongs to the essential aspects of heterogeneous photocatalysis. First, the efficacy of six lamps with various radiation intensity and distribution characteristics is contrasted. The topic of discussion is the photocatalytic hydrogen production from a methanol-water solution in the presence of a NiO-TiO_2_ photocatalyst. The second section is focused on the potential of a micro-photoreactor system–the batch reactor with a micro-reactor with a circulating reaction mixture, in which the photocatalytic reaction takes place using TiO_2_ immobilized on borosilicate glass. Continuous photocatalytic hydrogen generation from a methanol-water solution is possible in a micro-photoreactor. This system produced 333.7 ± 21.1 µmol H_2_ (252.8 ± 16.0 mmol.m^−2^, the hydrogen formation per thin film area) in a reproducible manner during 168 h.

## Graphical Abstract

**Figure:**
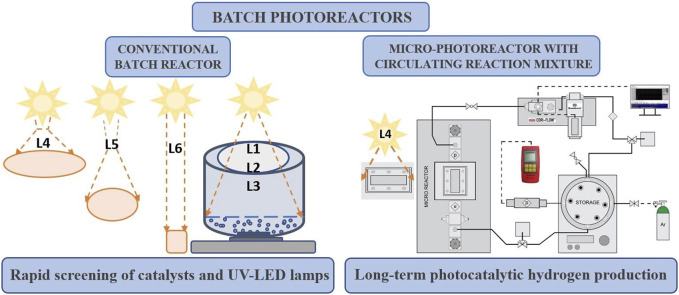

## 1 Introduction

For hydrogen production, photocatalytic water-splitting (Ahmad et al., 2015; Ng et al., 2021; Villa et al., 2021) is a promising way to convert solar energy into clean energy (Fujishima and Honda, 1972). Numerous studies comparing photocatalytic hydrogen production using various photocatalyst types have been reported [for example, see Refs (Maeda, 2011; Sakata et al., 2015; Chen et al., 2019; Rafique et al., 2020)]. TiO_2_-based photocatalysts are the most widely used and efficient photocatalysts (Do et al., 2020; Li et al., 2020; Dharma et al., 2022). Nowadays, employing heterojunction photocatalysts to produce hydrogen is alluring and very attractive; one such catalyst is NiO-TiO_2_, which demonstrated intriguing results (Yu et al., 2015).

Photocatalytic hydrogen production can be affected not only by the type of photocatalyst and its properties but also by many other factors, such as the composition of the reaction mixture, reaction conditions, irradiation source (wavelength, intensity in reaction volume, irradiation distribution), and reactor type (Ahmed et al., 2011; Amakiri et al., 2021; Enesca, 2021). The irradiation source is a significant factor in photocatalysis, as photon transfer or low quantum efficiency are still significant problems in photocatalytic applications. To circumvent some of the current limitations, other reactor designs and operating conditions have been proposed (Deba et al., 2023).

In the case of heterogeneous photocatalysis, the required energy source depends on the band gap energy of the photocatalyst [e.g., for TiO_2_ λ ≤ 387.5 nm (Schneider, 2015)]. The fundamental physical principle of all irradiation sources is the luminescence of excited atoms or molecules, where the electron is returned from the excited higher states to the ground state with simultaneous irradiation emissions. Based on operating principles, lamps can be divided into arc, incandescent, fluorescent, and lasers (Pareek, 2005). Ideally, using sunlight containing approximately 3%–5% ultraviolet irradiation would be desirable. Thus, mercury lamps and solar light are used in the research and application of photocatalysis. Mercury is a toxic, rare metal that burdens the environment. This limitation is associated with the end of the lamp’s life. As a mercury lamp only runs for approximately 1,500–20,000 h (Purpura et al., 2014), UV-LED lamps have inherently long lifetimes (Rasoulifard et al., 2011; Purpura et al., 2014), typically up to tens of thousands (50,000–80,000 h) of operating hours. The most significant advantage of using a UV-LED lamp is the substantial energy savings.

LEDs are also affordable and highly efficient irradiation sources, cheap, compact, lightweight, and have a lower operating temperature than conventional light sources. UV LEDs are also irradiation sources capable of producing monochromatic light with a narrow emission spectrum. In recent years, the electrical efficiency of UV-LED lamps has increased to values between 40% and 50%, which is significantly higher than traditional mercury vapor lamps and is rapidly approaching visible LEDs. Their efficiency is expected to increase further in the coming years if their development is comparable to that of visible light LEDs, greatly improving their photocatalytic water treatment efficiency (Tokode et al., 2014; Martín-Sómer et al., 2023). In addition, they can be turned on and off instantly (no warm-up) and do not contain harmful materials (Almquist et al., 2022).

Many research groups have already studied the influence of lamp intensity on photocatalytic efficiency (Pansamut et al., 2013; Kumar and Pandey, 2017; Wang et al., 2020; Amakiri et al., 2021). In most cases, the authors reported that hydrogen production increased with the increasing intensity of irradiation used (Baniasadi et al., 2013; Bell et al., 2013; Cheng et al., 2018). However, higher light intensities can cause much more significant energy losses instead of favoring the degradation of organic compounds. The key is determining the appropriate irradiation intensity to reduce the energy loss due to charge carrier recombination and ensure reproducibility in all measurements at different times (Amakiri et al., 2021). In general, in the case of heterogeneous reactions, it is observed that in some cases, the dependence of the reaction rate on the irradiation intensity becomes nonlinear, and the yields begin to decrease gradually (Bloh, 2019). However, more attention should be focused on comparing commercially available lamps and their illumination distribution and profile. The choice of an appropriate light source is also a crucial design factor for the efficient excitation of photogenerated species, which results in the creation of active radical species. Furthermore, the placement of the light source in the photocatalytic reactor impacts the effectiveness of the light received by the photocatalyst. Because of the high surface-to-volume ratios in the micro-photoreactor, the illuminated surface area is an important design parameter (Shukla et al., 2021). Photocatalytic water splitting is most often performed in two basic reactor configurations depending on the deployed state of the photocatalyst (powder form suspended in liquid and photocatalyst immobilized onto continuous inert carriers).

Most authors use batch reactors and powder photocatalysts in water-splitting reactions (Xing et al., 2013; Azam et al., 2019; Deng et al., 2019; Fajrina and Tahir, 2019; Guba et al., 2019; Visan et al., 2019; Toe et al., 2022; Sarkar et al., 2023). Its advantage is the possibility of using an external irradiation source during the reaction, excellent irradiation distribution in the entire volume of the reaction mixture, good mass transport, and a large specific area of the photocatalyst concerning the reactor volume. On the other hand, separating photocatalyst particles from the reaction mixture is very difficult, and there is a limited depth of irradiation transmission. The distribution of light intensity depends on whether the lamp is along a reactor (cylindrical shape) (Enesca, 2021) or is located at the top of the reactor (Sergejevs et al., 2017). Another disadvantage is that the reaction products remain in contact with the metal-doped photocatalyst particles for longer, promoting an undesired back reaction (Reilly et al., 2012). The unwanted back reaction is predominant in suspended photocatalyst suspensions because the H_2_ and O_2_ produced remain in contact with the high surface area photocatalyst particles for an extended period (Reilly et al., 2018).

Monolithic reactors (Lin and Valsaraj, 2006; Tahir and Amin, 2013; Maroto-Valer and Ola, 2015; Fajrina and Tahir, 2019), fixed bed fluidized bed reactors (Tahir and Amin, 2013; Vaiano et al., 2015; Fajrina and Tahir, 2019), optical fibers (Lin and Valsaraj, 2006; Tahir and Amin, 2013; Maroto-Valer and Ola, 2015), or reactors with photocatalysts immobilized in thin film (Oelgemoeller, 2012; Sambiagio and Noël, 2020) represent other attractive systems possessing easy separation and re-usability of photocatalysts. Immobilized thin-film photocatalysts are limited by poor light distribution, limited mass transfer, and a small photocatalyst surface area relative to reactor volume (Belver et al., 2019). However, they offer the convenient handling of the photocatalyst and reduce the risk of backreaction because the reaction products and the metal-doped photocatalyst are easily separated.

The significant advantage of immobilized photocatalysts is that they eliminate the need for separate catalysts while preventing photocatalyst agglomeration and deactivation (Mei et al., 2023). Micro-reactors using photocatalysts immobilized in thin film ensure an even light distribution due to their small size, short optical path, and large area-to-volume ratio. Due to the high density of photons in microreactors, it is clear that fast reaction times are required compared to a conventional batch reactor (Adamu et al., 2020). Properties unique to microreactors include laminar flow, short molecular diffusion distances, large specific interfacial areas, and excellent heat transfer characteristics. Particularly in photochemical reactions, microreactors show higher homogeneity of spatial illumination and better light penetration over the entire reactor depth than large-area reactors (Rashmi Pradhan et al., 2019; Zhan et al., 2020). Although external mass transport limitations are often considered for microreactors, internal (porous) diffusion limitations are only occasionally discussed (Zhan et al., 2020).

In this manuscript, we have focused our attention on comparing the efficiency of using six different lamps, which differed not only in the intensity of irradiation and their power but also in the profile and distribution of irradiation inside the batch photoreactor. In this part, NiO-TiO_2_ powder photocatalyst is studied with very low NiO loading (0.2 wt% NiO), which is expected to improve the photocatalytic behavior of pure TiO_2_. The second part is focused on a micro-photoreactor system–a batch reactor with a micro-reactor through which that reaction mixture is circulating. A micro-photoreactor uses a photocatalyst immobilized in a thin film on the glass. In this part, pure TiO_2_ thin film is used as a photocatalyst. TiO_2_ photocatalysts could be taken as standards to which the photocatalytic activity is usually compared, especially regarding a new design or type of process or reactor. In this manuscript, we focused mainly on the continuity of the entire process, reproducibility, stability, and evaluation of the contribution of the given micro-photoreactor.

## 2 Experimental

### 2.1 Preparation of photocatalyst

*Powder NiO-TiO*_*2*_ *photocatalyst.* 0.2 wt% NiO-TiO_2_ was prepared by the sol-gel method in a reverse micellar environment [for details, see Kočí et al. (Kočí et al., 2018)].

*The thin film of TiO*_*2*_ *photocatalyst.* The transparent sol of TiO_2_ was prepared by the sol-gel method using the reverse micellar environment. The molar ratio of individual chemicals forming the titania sol was following: cyclohexane: Triton X-114: distilled water: titanium (IV) isopropoxide = 11: 1:1:1. After preparation of the sol, it was left to stand for 24 h. After that, one or two TiO_2_ were deposited on degreased and washed dry borosilicate glass. The dip-coating method realized on the Coater 5 STD (from idLab s.r.o., ČR) was used for layer deposition from both sides of glasses, using the following parameters: the immersion velocity of 150 mm.min^−1^, delay in the sol 60 s, and the emerging velocity of 60 mm.min^−1^. After the emerging of the glass, the layers on one side were immediately removed by cyclohexene. After 4 h drying on air, the layers were calcined at 400°C with a temperature ramp of 3°C.min^-1^ for 4 h.

### 2.2 Characterization of photocatalysts

Raman spectra of NiO-TiO_2_ powder and TiO_2_ thin film were measured on a Nicolet DXR SmartRaman spectrometer (Thermo Fisher Scientific, United States).

The properties of NiO-TiO_2_ powder photocatalyst are described in Supplementary Material S1—structural properties results from X-ray diffraction analysis (Supplementary Figure S1, Supplementary Table S1), Raman spectroscopy (Supplementary Figure S2) and Diffuse reflectance UV-Vis spectroscopy (Supplementary Figure S3, Supplementary Table S1).

### 2.3 The properties of TiO_2_ thin film

Elemental analysis and mapping of the TiO_2_ thin film (1 layer) sample and the borosilicate glass on which the sample is deposited was measured on a scanning electron microscope (LYRA3, Tescan) equipped with EDX analyzer (AZtec X-Max 20, Oxford Instruments) at an acceleration voltage of 5 and 20 kV. TiO_2_ thin film sample was coated with 20 nm of carbon in the Leica EM ACE200 instrument.

The thickness of the TiO_2_ thin film was determined using spectroscopic ellipsometry using a J.A.Woollam VASE instrument in the spectral range 250–1700 nm for incidence angles of 50, 60, and 70° and 0°.

UV-Vis measurement of TiO_2_ thin film was measured on Cintra 2020 (GBC Scientific equipment, Australia), and indirect band gap energy was determined from the dependence of (α·h·ν)^1/2^ against photon energy.

### 2.4 Photocatalytic hydrogen generation from methanol/water solution

Two different types of reactors were used: a conventional batch reactor and a micro-photoreactor–batch photoreactor with a circulating reaction mixture.

#### 2.4.1 Batch reactor

**FIGURE 1:**
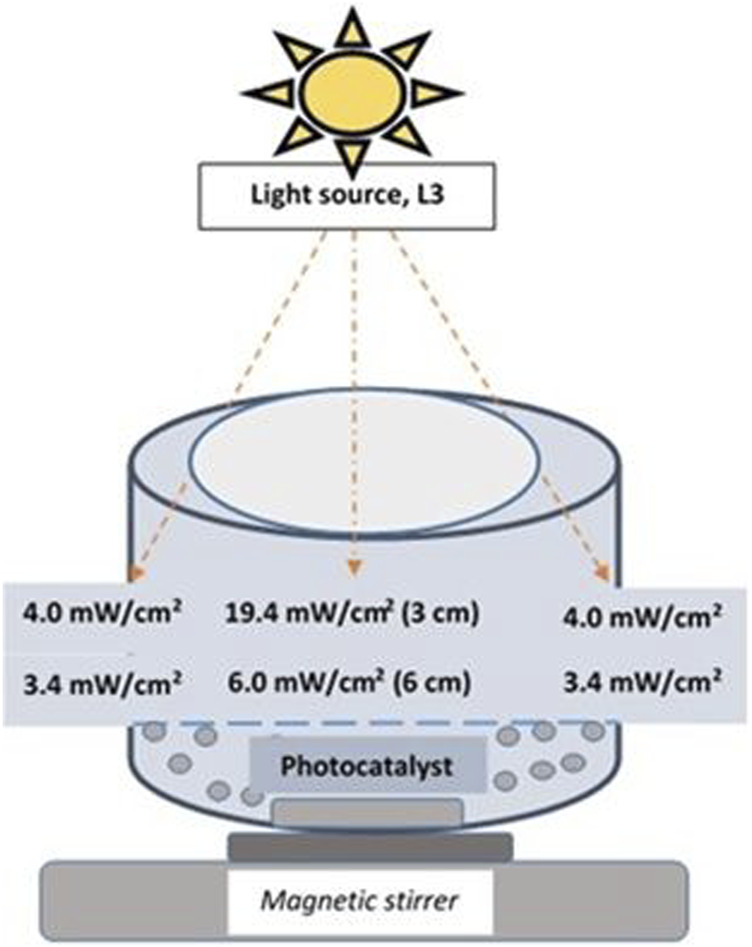
Graphical representation of the batch reactor using a UV-LED Solo P lamp.

The photocatalytic water-splitting was carried out in a batch photoreactor (Figure 1) made of stainless steel. 100 mg of the powdered NiO-TiO_2_ photocatalyst (diameter of 0.16–0.25 mm) was added to 100 mL of an aqueous methanol solution (50 vol% of methanol). The reaction mixture was continuously stirred at 350 rpm. The upper part of the reactor was provided with an opening made of quartz glass. The distance between the irradiation source and the level of the reaction mixture was 6 cm. All irradiation sources used are listed in Table 1 and described in more detail below. Before the photocatalytic reaction, the reactor was purged with argon to remove air from the reaction mixture. The reaction was measured at room temperature. At the beginning of the reaction, the argon pressure above the reaction mixture was around 160 kPa. The pressure of gas slightly increased during a reaction due to the formation of hydrogen. The pressure was measured by a digital barometer (Greisinger GRS 3100). A sample of hydrogen gas was taken every hour through a gas-tight syringe. The gaseous products were analyzed by a gas chromatograph (7890B GC System, Agilent Technologies, United States) equipped with a TCD (thermal conductivity detector) and argon as a carrier gas. All photocatalytic tests in the batch reactor were measured repeatedly.

**TABLE 1:** Properties of used lamps.

Type of lamp	Sign	Wavelength	Intensity^a^ (mW.cm^−2^)	Power (W)	Emission surface^b^ (cm)	Recommended working distance^c^
Pen-Ray UV mercury lamp (Analytic Jena, United States)	L1	365	0.1	8	> reactor diameter	not specified
Mid Power Mounted LED (ThorLabs, Germany)	L2	365	2.0	2	> reactor diameter	not specified
UV-LED Solo P without optics (Opsytec Dr. Gröbel, Germany)	L3	365	6.0	5	> reactor diameter	not specified
UV-LED Solo P—high power (Opsytec Dr. Gröbel, Germany)	L4	365	7.0	5	> reactor diameter	ca 0.7 cm
UV-LED Solo P—standard (Opsytec Dr. Gröbel, Germany)	L5	365	50	5	diameter 4 cm circular profile	ca 2 cm
UV-LED Solo P—parallel beam (Opsytec Dr. Gröbel, Germany)	L6	365	205	5	1.5 × 1.5 square profile	3–6 cm

^a^ Intensity from a distance of 6.0 cm (distance to the liquid surface).

^b^ Reactor diameter is 8 cm.

^c^ Recommended working distances given by the company.

##### 2.4.1.1 Commercially available lamps for photocatalytic applications

**FIGURE 2:**
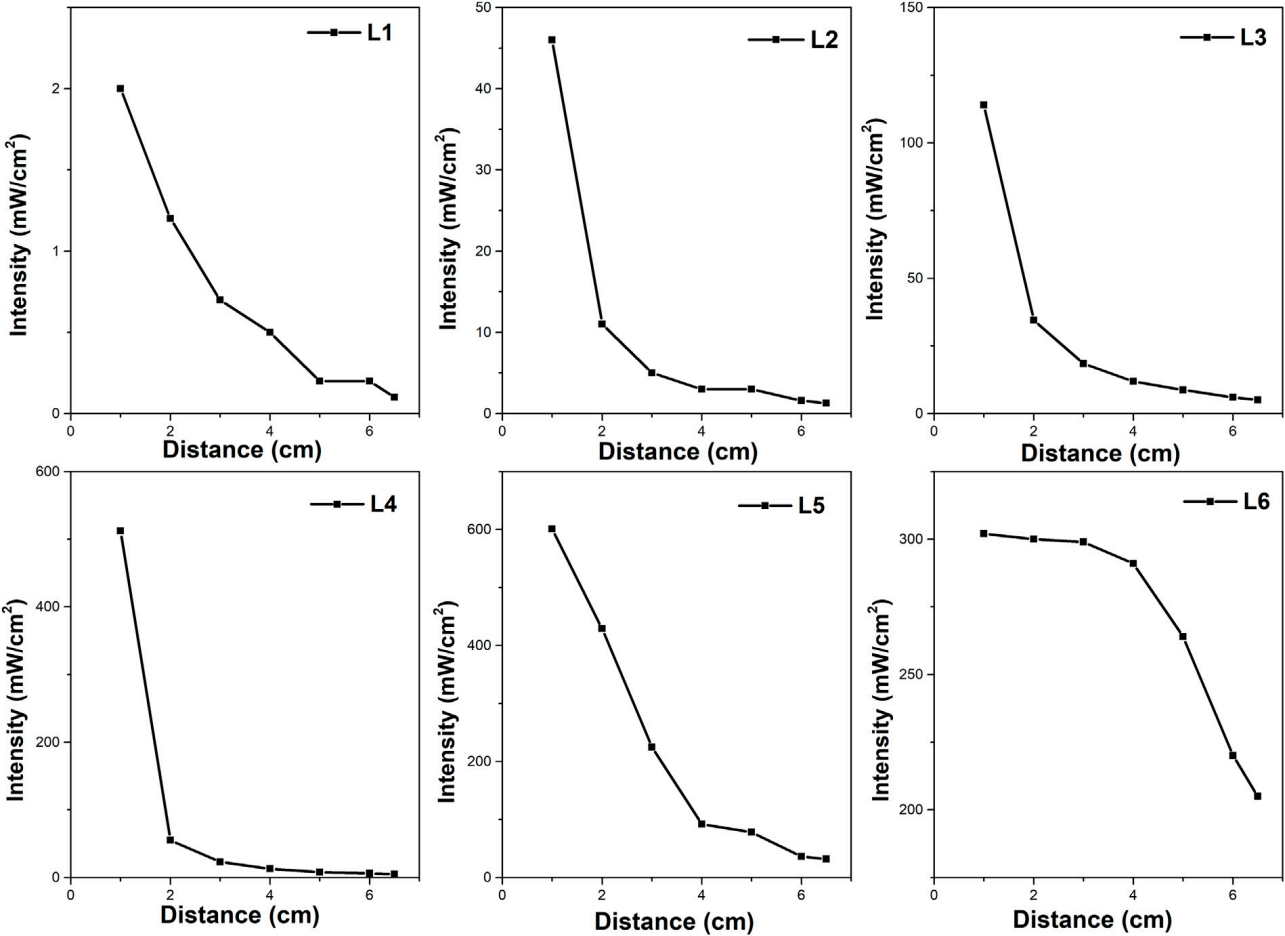
Dependence of the lamp intensity on the distance from the irradiation source (measured perpendicular to the source).

Table 1 shows the properties of the six lamps used in the study. All UV LED lamps used in the photocatalytic experiment had a wavelength of 365 nm; only one (mercury lamp) falls under the category of polychromatic light radiation. Figure 2 shows the dependence of their intensities at different distances (from 1 to 6 cm), while the distance of the light source from the reaction mixture was 6 cm for all experiments. Lamp intensities were measured with a radiometer (Radiometer RM-12, Opsytec Dr. Gröbel, Germany) calibrated at 365 nm. The measurement of the radiation intensity of the lamps was carried out in a quartz crucible in which the height of the reaction mixture was equivalent to that in the reactor, and the radiometer sensor was placed under this crucible (the photocatalyst was not put in the liquid during the intensity measurement in this case). Lamps L1 (Pen-Ray UV mercury lamp, Analytic Jena, United States) and Lamps L2 (Mid Power Mounted LED type M365LP1, ThorLabs, Germany) have an irradiation profile with an exposure area higher than the reactor area (diameter greater than 8 cm). The Mid Power Mounted LED (L2) manufacturer reported a lamp intensity of 2.1 mW.cm^−2^ from a distance of 20 cm. L3 (UV-LED Solo P, Opsytec Dr. Gröbel, Germany) has an irradiation profile with an exposure area higher than the reactor area (diameter greater than 8 cm).

For the UV-LED solo P lamp designated as L3 (Opsytec Dr. Gröbel, Germany) (Figure 1), several beam profiles were available with interchangeable optics (Supplementary Figure S4), i.e., high power (L4) (Supplementary Figure S4A), standard (L5) (Supplementary Figure S4B), and parallel optics (L6) (Supplementary Figure S4C). High power (L4) and standard (L5) optics best suit small point diameters. Larger working distances and point diameters can be achieved with parallel beam (L6) optics. If no optics are used, the space in the photoreactor is illuminated equally, and the irradiation is not focused only on a certain point in the reactor. The maximum possible intensity of the UV-LED solo P lamp specified by the manufacturer can be up to 25,000 mW.cm^−2^. L3 and L4 have an irradiation profile with an exposure area higher than the reactor area. On the other hand, L5 has a circular irradiation profile with a diameter of about 4 cm from a distance of 6 cm (distance from the top of the lamp to the liquid surface), less than the diameter of the photoreactor. The L6 lamp also provides a square irradiation profile with an area of about 1.96 cm^2^ from a distance of 6 cm.

#### 2.4.2 Micro-photoreactor using photocatalyst in the form of a thin film

**FIGURE 3:**
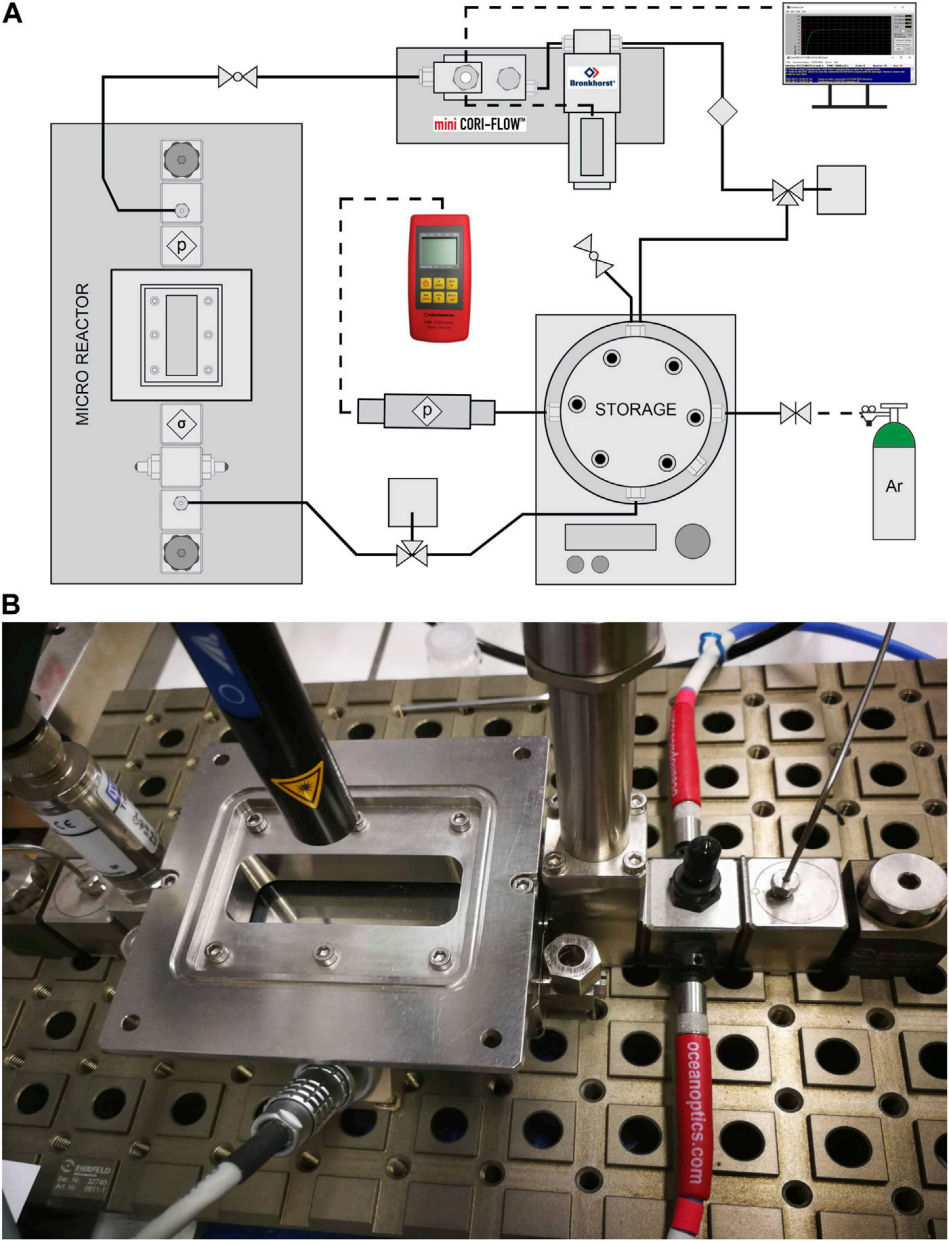
**(A)** Scheme of flow micro-photoreactor and **(B)** detailed photo of the main part of the system–microreactor.

Long-term experiments (168 h) of the photocatalytic decomposition of an aqueous methanol solution were measured in a micro-photoreactor respectively batch photoreactor with a circulating reaction mixture. The entire micro-photoreactor system (Figure 3A) consists mainly of a steel microreactor (Figure 3B), in which there is a glass made of Borofloat glass with a thin film of TiO2 photocatalyst, which was irradiated from above by UV-LED solo P lamp (Opsytec Dr. Gröbel, Germany) with high power optics (L4). The photocatalytic reaction takes place at this location (microreactor, Figure 3), and the irradiated area of the sample was 1,320 mm^2^ from a distance of 4.5 cm from the radiation source (in this case, the intensity of irradiation is 13 mW.cm-2). This main part of the micro-photoreactor from Ehrfeld, Germany, was equipped with a pressure sensor. The reaction mixture flows from the storage through the microreactor at a rate of 6.4 mL.min^−1^ thanks to a digital mass flow meter/controller for liquids and gases mini Cori-Flow equipped with digital control software FlowDDE and FlowPlot (Bronkhorst, Netherlands) to the storage. The volume of the entire system of micro-photoreactor is approximately 130 mL, and most of this volume was located in stainless-steel storage space. The reaction mixture was in a stainless-steel storage space equipped with a pressure sensor, an inert gas inlet and outlet, a gas sampling septum, an inlet and outlet for continuous recirculation flow of the liquid reaction mixture through the hole micro-photoreactor system, and a by-pass for easy cleaning of the entire reactor.

Before the reaction, the entire micro-photoreactor was rinsed with 50% methanol solution and then charged with 50 mL of the reaction mixture (50 vol% of methanol). Furthermore, during the flow of the reaction mixture, the reaction mixture was purged with inert gas (argon) due to the storage space of the reaction mixture, which takes place for 30 min. After rinsing the reaction, the whole system was pressurized to approximately 170 kPa and allowed to stabilize. After stabilization, the reaction was switched on by switching on the UV-LED solo P lamp with high power optics (L4) in the micro-photoreactor area (Figure 3), and the reaction runs for a total of 7 days (168 h) when a sample was taken twice with a 500 µL gas-tight syringe every 24 h, a total of 6 times in 7 days.

## 3 Results and discussion

### 3.1 Batch reactor - Evaluation of commercially available lamps for photocatalytic applications

**FIGURE 4:**
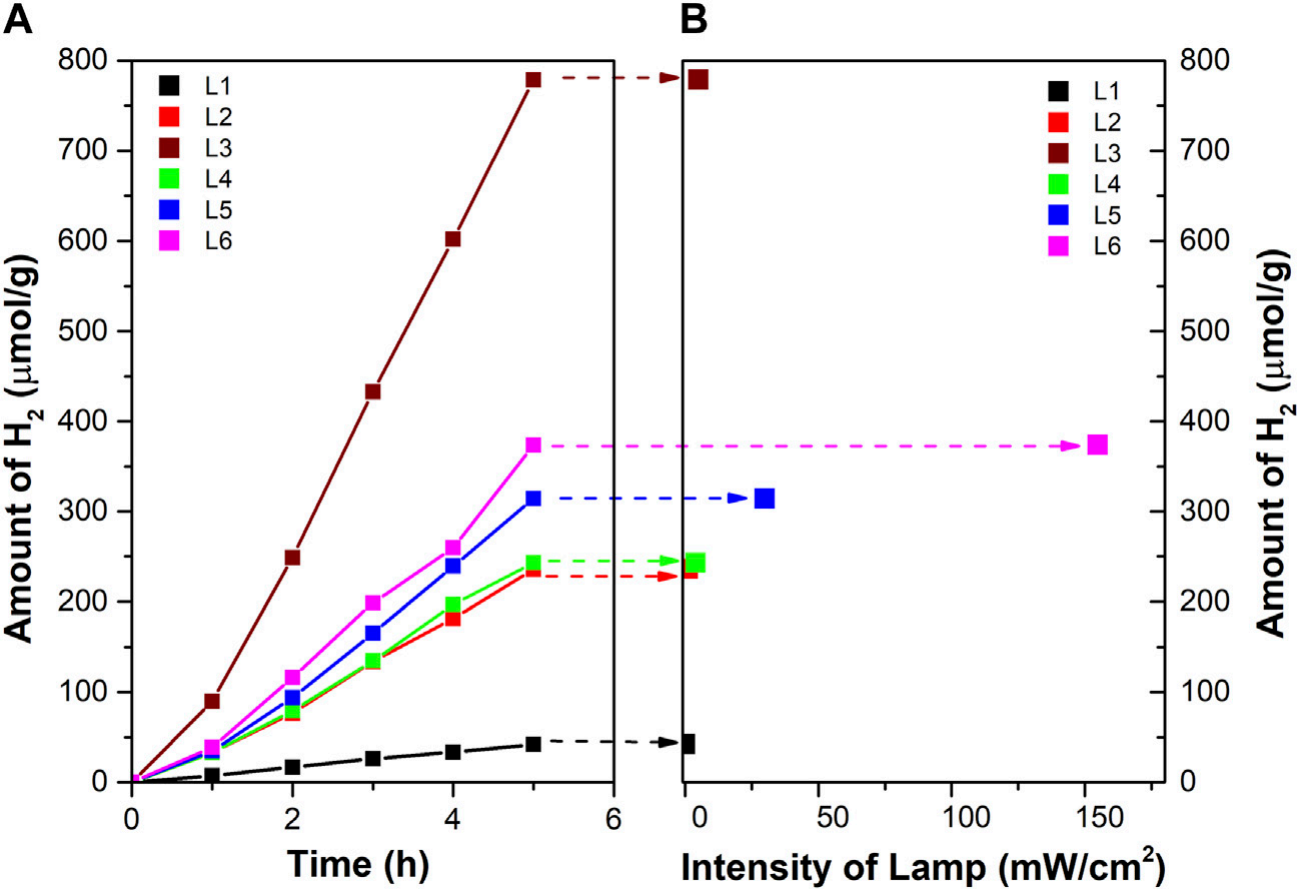
**(A)** Amount of hydrogen for different types of the lamp in a batch photoreactor and **(B)** dependence of radiation intensity on hydrogen production after 5 h of irradiation.

Figure 4A shows the amount of hydrogen produced in a batch reactor as a function of reaction time for each of the six different lamps described in the experimental part (codes L1 to L6). The lamps differ in intensity (mW.cm^−2^, Table 1) and the distribution of irradiation inside the space of the reactor (Figure 1, Supplementary Figure S4; Figure 2). All used LED lamps have the same nominal wavelength of 365 nm. The amount of formed hydrogen increased in the order of UV-LED solo P without optics (L3) > UV-LED solo P–parallel beam (L6) > UV-LED solo P–standard (L5) > UV-LED solo P–high power (L4) ≈ Mid Power Mounted LED (L2) > Pen-Ray UV mercury lamp (L1).

Figure 4B shows the dependence of the amount of hydrogen on the intensity (mW.cm^−2^) of the lamp at a time of 5 h and a 6.0 cm distance from the source of irradiation (distance to the liquid surface in the reactor, Table 1; Figure 2).

It should be mentioned that the rate of the photocatalytic reaction is directly proportional to the intensity of the irradiation only at a low irradiation intensity. At a higher irradiation intensity, it is already proportional to the square root of the irradiation intensity and is already independent of the irradiation intensity value (Ollis et al., 1991; Yang and Liu, 2007; Hisatomi et al., 2015). This is due to possible energy losses caused by the rapid recombination of e^−^/h^+^ pairs (Hisatomi et al., 2015).

Firstly, the L3, L4, L5, and L6 lamps originate from the same source of radiation but differ in their optics, leading to the different radiation profiles of individual lamps (the intensity and the distribution of radiation in the space of the reactor, Figure 1 and Supplementary Figure S4). The highest amount of hydrogen was produced by using an L3 lamp. However, this lamp did not possess the highest radiation intensity at a 6.0 cm distance from the source of radiation (Figure 2). Both, UV-LED solo P–standard (L5) and UV-LED solo P–parallel beam (L6) possessed higher radiation intensities at a 6.0 cm distance from the source of radiation in contrast to UV-LED solo P without optics (L3). However, these lamps have an optimal radiation radius smaller than the reactor’s radius, from which it can be suggested that the radiation intensity at the reactor walls is lower. This reason is probably connected to the better utilization of radiation by less intense lamps on the sides of the reactor than in the case of lamps with higher radiation intensity in the greater depth of the reaction mixture. At the same time, the already mentioned changing dependence of the reaction rate on the radiation intensity can also contribute to the lower formation of hydrogen. As part of the lamp comparison, unfortunately, we could not provide a 3D model of the radiation intensity in the volume of the whole batch reactor.

Secondly, the L1 and L2 lamps possessed the lowest amount of hydrogen produced. Both these lamps possessed a radiation diameter higher than the diameter of the reactor. Still, these lamps had the lowest radiation intensity, a 6.0 cm distance from the source of radiation, which eventually led to a lower amount of hydrogen produced.

This example shows the contribution of lamp radiation distribution to its photocatalytic performance, and it partially explains the origin of different photocatalytic performances reported by other laboratories for close photocatalysts. Even though LED lamps have a higher purchase price, they are increasingly replacing traditional medium-pressure mercury-based lamps, mainly due to their long life, low consumption, and better placement of more lamps in the reactor.

### 3.2 Micro-photoreactor

Efficient radiation contact with the reaction mixture is one of the essential parameters of photocatalytic activity. Thus, we constructed the micro-photoreactor system—batch reactor with a micro-reactor with the circulating reaction mixture (Figure 3). Compared to conventional reactors, reactors with continuous motions of the reaction mixture are more specific in photocatalytic reactions due to the light irradiation requirements (Manassero et al., 2023). Thus, based on the study in the first part of the manuscript, a lamp with high-power optics (L4) was evaluated as optimal for further experiments in a micro-photoreactor. The requirements for the irradiated portion of the microreactor, where the photocatalytic reaction takes place, were mainly the irradiation of the entire surface of the microreactor and, at the same time a high intensity near the source, given that the reaction takes place on an immobilized thin film on borosilicate glass, which is in contact with the laminar film of the reaction mixture, and there is no need uniformly irradiated to depth. In addition, lamp L4 can illuminate the entire glass with an immobilized photocatalyst from a lower distance than it was used in the batch reactor measurement. Also, the lamp L4 seafront is focused on a shorter working distance. This reactor enables effective radiation contact only in a small volume of the reaction mixture passing through the micro-photoreactor.

#### 3.2.1 TiO_2_ photocatalyst in the form of a thin film

The thin film of the TiO_2_ layer is formed by anatase modification of TiO_2_, as it is evidenced by the Raman spectrum (Supplementary Figure S5). Raman spectrum of TiO_2_ thin film shows maxima of bands at 144, 397, 517, and 639 cm^−1^ which correspond to anatase modification of TiO_2_ (Supplementary Figure S5). The thickness of the TiO_2_ thin film on borosilicate glass is 232 ± 2 nm (spectroscopic ellipsometry). The band gap energy of TiO_2_ thin film is 3.4 eV (Supplementary Figure S6).

To analyze TiO_2_ thin film and at the same time the borosilicate glass, there was a deliberately created groove in the TiO_2_ thin film. Supplementary Figure S7 shows the mapping of the thin TiO_2_ film obtained from SEM-EDX. The uniform distribution of titanium on the surface of the entire material is visible beyond the mentioned groove. Supplementary Table S2 gives the composition of TiO_2_ thin film and the borosilicate glass (B, O, Si) with a small amount of Na, K, and Al. Since the thickness of TiO_2_ thin film is shallow, the composition of TiO_2_ thin film involves both TiO_2_ and elements of the borosilicate glass.

Supplementary Figure S8A shows a scratch in the TiO_2_ thin layer, from which the profile of the TiO_2_ particles in the thin layer is noticeable. Supplementary Figure S8B shows the homogeneous distribution of the TiO_2_ in the thin film.

#### 3.2.2 Long-term experiments

**FIGURE 5:**
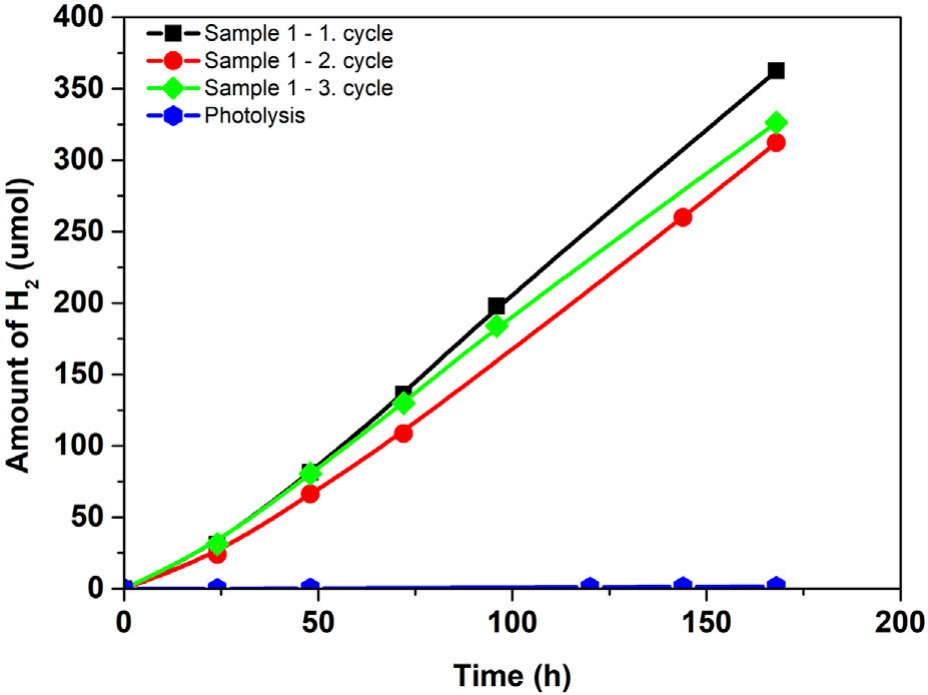
Amount of hydrogen in photocatalytic hydrogen production from methanol-water solution in micro-photoreactor.

Figure 5 shows the dependence of the amount of hydrogen formed from water-methanol solution in a micro-photoreactor system using the photocatalyst in the form of a thin film. This configuration is attractive due to the absence of the process of photocatalyst separation, as it is an immobilized photocatalyst applied on thin glass. The sample can be easily removed without damage from the micro-photoreactor and used repeatedly. The reproducibility of the repeated use of the photocatalyst is shown in Figure 5 (Sample 1-1.-3. cycle). The TiO_2_ sample 1 has been used in a total of three cycles (Figure 5 Sample 1-1.-3. cycle), which showed hydrogen production of 333.7 ± 21.1 µmol (i.e., 252.8 ± 16.0 mmol.m^−2^, the formation of hydrogen per area of the thin film), this is a total of three times the reuse of the same photocatalyst. It is clear that over the first three 168-h photocatalytic cycles, the amount of hydrogen is roughly constant. The immobilized photocatalyst can be utilized again in this configuration, making it ideal for long-term continuous studies. In addition, the photolysis of glass measured without an immobilized photocatalyst indicated 1.5 µmol (i.e., 1.14 mmol.m^−2^). of hydrogen generation after 168 h of reaction. It demonstrates photolysis’s insignificant role in the photocatalytic activity of the immobilized TiO_2_ catalyst.

The advantage of a micro-photoreactor is that thanks to the recycling system and the subsequent continuity of the entire procedure, it is configured for long-term research as well as for maximum utilization of the reaction mixture. According to Figure 3, it is evident that after a photocatalytic reaction in the micro-reactor space, the mixture goes back to the storage and returns to the system. In the storage, there is sufficient space for both liquid and gaseous phases, so the gaseous phase should be focused on this space when passing through the entire reactor. That´s why we can analyze gas hydrogen from the reaction mixture like in the conventional batch reactor. The micro-photoreactor also achieves stable/reproducible results in long-term experiments—after three consecutive cycles (three repeating cycles each of 168 h).

It is hard to compare the reported data with the literature as scientific groups use different configurations of micro-photoreactors, types of lamps, and sacrificial agents. Miquelot et al. (Miquelot et al., 2019) published H_2_ production using anatase TiO_2_ that increased from 4.4 to 78.9 mmol.m^−2^ (cumulative value after 66 h, reaction mixture of ethanol-water 1:1, 300 W Xe-lamp), where the H_2_ formation enhanced due to was published to be enhanced via increasing T_d_ induces a substantial increase in morphological complexity. If we compare the amount of hydrogen production we achieved at the same reaction time, we obtain 86.55 ± 7.99 mmol.m^−2^ (66 h), comparable to the published value of 78.9 mmol.m^−2^. However, it should be noted that both results were achieved under different conditions. For example, while we are using a methanol-water solution and 5 W LED lamp, the published result was obtained instead of ethanol-water solution and using a 300 W Xe lamp as a light source. We use TiO_2_ photocatalyst prepared by the standard sol-gel method instead of the publication, where the photocatalysts with the unique orientation of TiO_2_ particles are used.

## 4 Conclusion

Scientific teams use different types of commercially available lamps in photocatalytic reactions. This work compared six different lamps, i.e., lamps with different optics, and thus the intensity of irradiation, their power, and the irradiation distribution inside the conventional batch photoreactor.

The highest yield of hydrogen was achieved with the L3 lamp (UV-LED Solo P, Opsytec Dr. Gröbel, Germany), which, although it has lower radiation energy at 6 cm from the source than the other two lamps (L5 and L6), nevertheless it distributes the radiation better in space.

Due to the different properties of all the lamps, the best lamp for both the conventional batch reactor (L3) and the micro-photoreactor (L4) was evaluated and selected. The contribution of that article is the introduction of very different properties of individual but commercially available lamps, which could find broad use depending on the application.

The micro-photoreactor–a batch reactor with a micro-reactor with the circulating reaction mixture offered the possibility of long-term generation of hydrogen from the methanol-water solution due to continuous recirculation of the reaction mixture using the immobilized photocatalyst for easy photocatalyst separation. In the micro-photoreactor, emphasis was placed on the possibility of a long-term continuous hydrogen generation process with excellent reproducibility and stability. The TiO_2_ thin film led to the production of 333.7 ± 21.1 µmol during 168 h (i.e., 252.8 ± 16.0 mmol.m^−2^, the formation of hydrogen per area of the thin film). The micro-photoreactor also achieves stable/reproducible results in long-term experiments - after three consecutive cycles (three repeating cycles each of 168 h).

## Data Availability

The original contributions presented in the study are included in the article/Supplementary Material, further inquiries can be directed to the corresponding author.
