# Supplementary material for: Role of lamp type in conventional batch and micro-photoreactor for photocatalytic hydrogen production
Source: Front Chem. 2023 Sep 20;11:1271410. doi: 10.3389/fchem.2023.1271410 (PMC10548134; doi:10.3389/fchem.2023.1271410)
Supplement: Supplementary file 1 [file DataSheet1.pdf]

Supplementary material

Characterization of NiO-TiO_2 photocatalyst

Experimental

X-ray diffraction (XRD) pattern was recorded using a diffractometer (MiniFlex 600, Rigaku, Japan) with a PDF-2 database and D/teX Ultra detector. The X-ray source was a CuKα tube operated at 40 kV and 15 mA. The slit width was set at 10 nm. The sample was measured at 10°/min at a step size of 0.02° and a 2θ range of 10–80°. The content of the phase was determined using the reference intensity ratio (RIR) method. (Dubnová, L., et al., Front. Chem. 9:803764. doi: 10.3389/fchem.2021.803764)

Raman spectra were measured on a Nicolet DXR SmartRaman spectrometer (Thermo Fisher Scientific, USA). (Kočí, K., et al., Front. Chem. 6:44. doi: 10.3389/fchem.2018.00044)

Diffuse reflectance spectrum was measured in quartz cuvettes using a GBS CINTRA 303 spectrometer (GBC Scientific Equipment, Australia) equipped with an integrating sphere. Kubelka–Munk function was calculated based on the equation $F(R\infty) = (1 - R\infty)^{2/}(2 \cdot R\infty)$, where R∞ is the diffuse reflectance from a semi-infinite layer. Indirect band gap energy was determined from the dependence of $(\alpha \cdot h \cdot \nu)^{1/2}$ against photon energy, where it is possible to assume that the absorption coefficient (α) is approximately equal to the Kubelka–Munk function. (Dubnová, L., et al., Applied Surface Science 469: 879-886. doi:10.1016/j.apsusc.2018.11.098)

Results

NiO-TiO_2 photocatalyst contain pure anatase phase based on XRD and Raman spectroscopy. Fig. S1 shows XRD pattern of 0.2 wt. % NiO-TiO_2 photocatalyst. Diffraction lines of material are evident at 2θ ≈ 25.3; 38.1; 48.1; 53.9; 55.1; 62.7; 69.2; 70.1; 75.2° reflecting (101), (004), (200), (105), (211), (204), (116), (220), (215) planes of the anatase phase (ICDD PDF, 00-064-0863). Table S1 gives the crystallite size and the lattice parameters of 0.2 wt. % NiO-TiO_2 photocatalyst. Fig. S2 shows Raman spectrum of 0.2 wt. % NiO-TiO_2 photocatalyst, where bands with the maximum at 144, 195, 396, 517, and 639 cm^{-1} correspond to the presence of the anatase phase. Fig. 3S shows DR UV-Vis spectrum and recalculated spectrum of 0.2 wt. % NiO-TiO_2 photocatalyst for determination of band gap energy. The band gap energy of the studied NiO-TiO_2 was 2.53 eV. (Table S1)

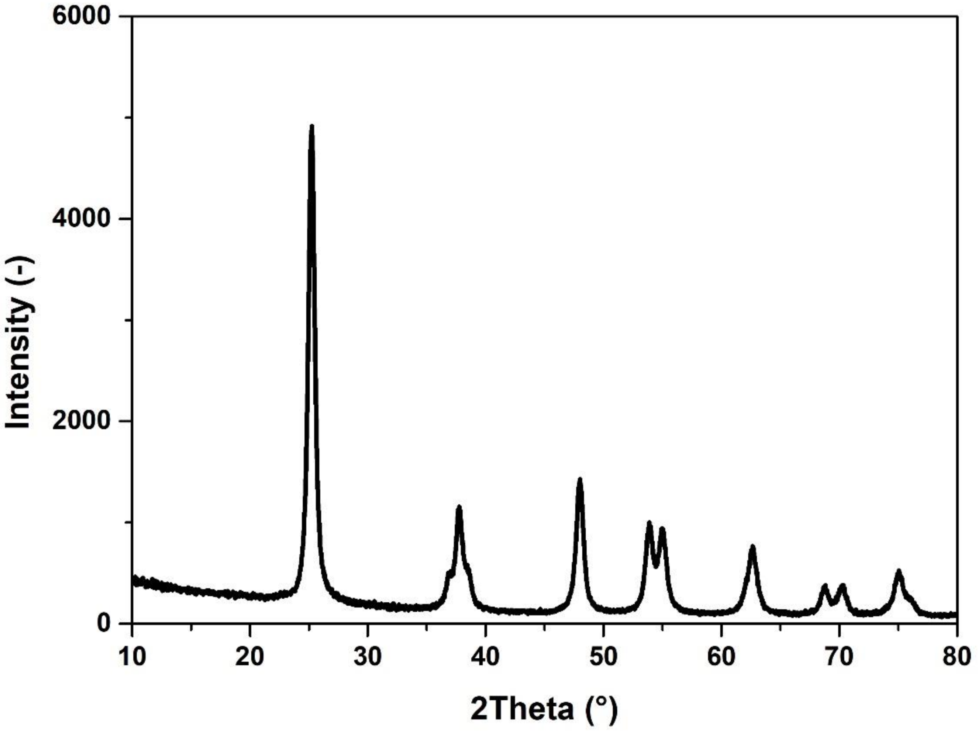

Fig. S1 X-ray diffraction analysis of 0.2 wt. % NiO-TiO_2 photocatalyst

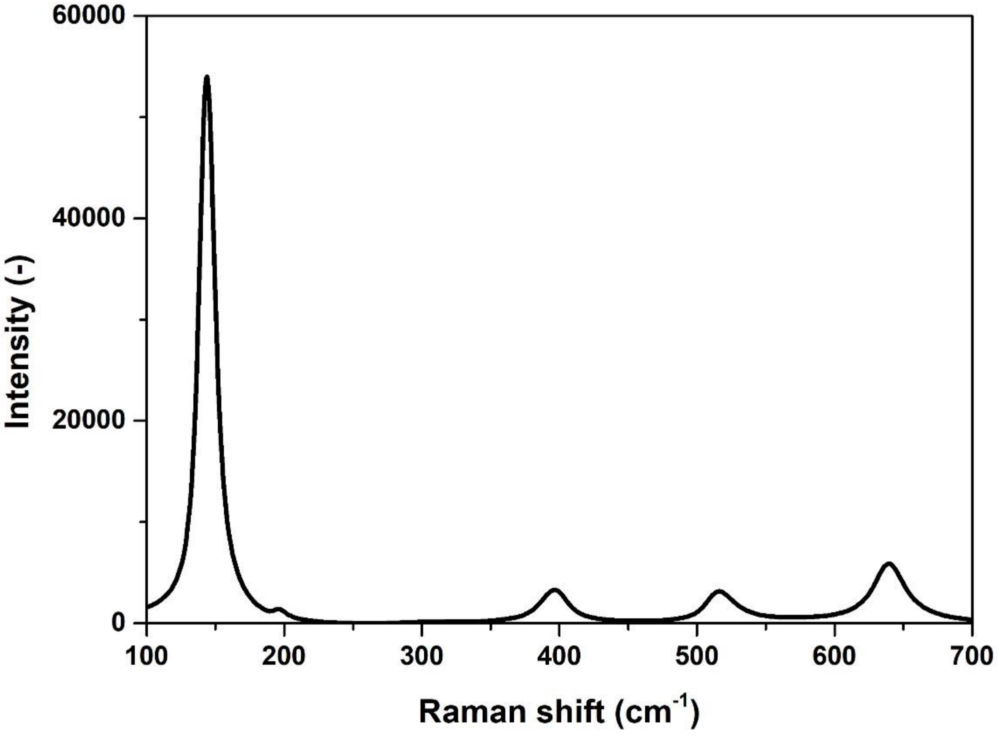

Fig. S2 Raman spectrum of 0.2 wt. % NiO-TiO_2 photocatalyst

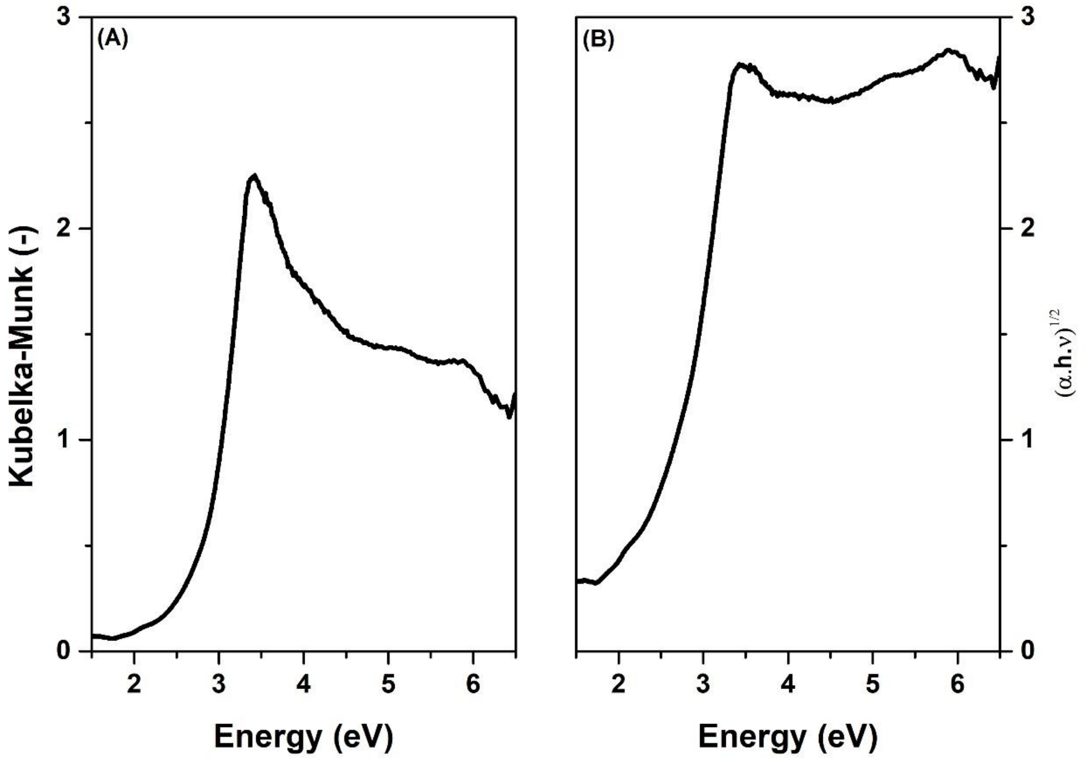

Fig. S3 DR UV-Vis spectrum (A) of 0.2 wt. % NiO-TiO_2 photocatalyst recalculated to dependence of $(\alpha \cdot h \cdot \nu)^{1/2}$ on energy (B).

Table S1 Lattice parameters, crystallite size, and indirect band gap of 0.2 wt. % NiO-TiO_2 photocatalyst

**Lattice parameters**		**Crystallite size**	**Anatase phase**	**Indirect band gap**
**a = b (Å)**	**c (Å)**	**(Å)**	**(%)**	**(eV)**
3,7921	9,5308	102	100	2.53

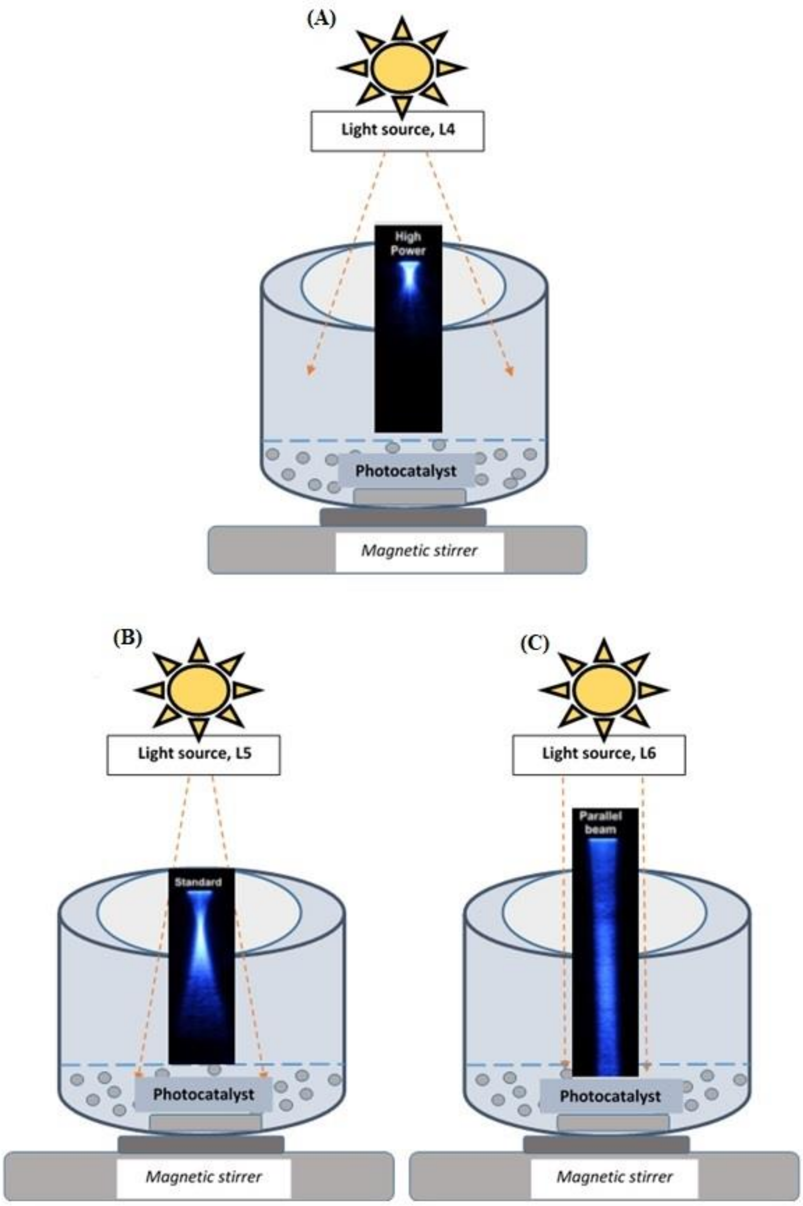

Fig. S4 Interchangeable optics and beam profiles of UV-LED solo P – high power (L4), UV-LED solo P – standard (L5) and UV-LED solo P – parallel beam (L6)

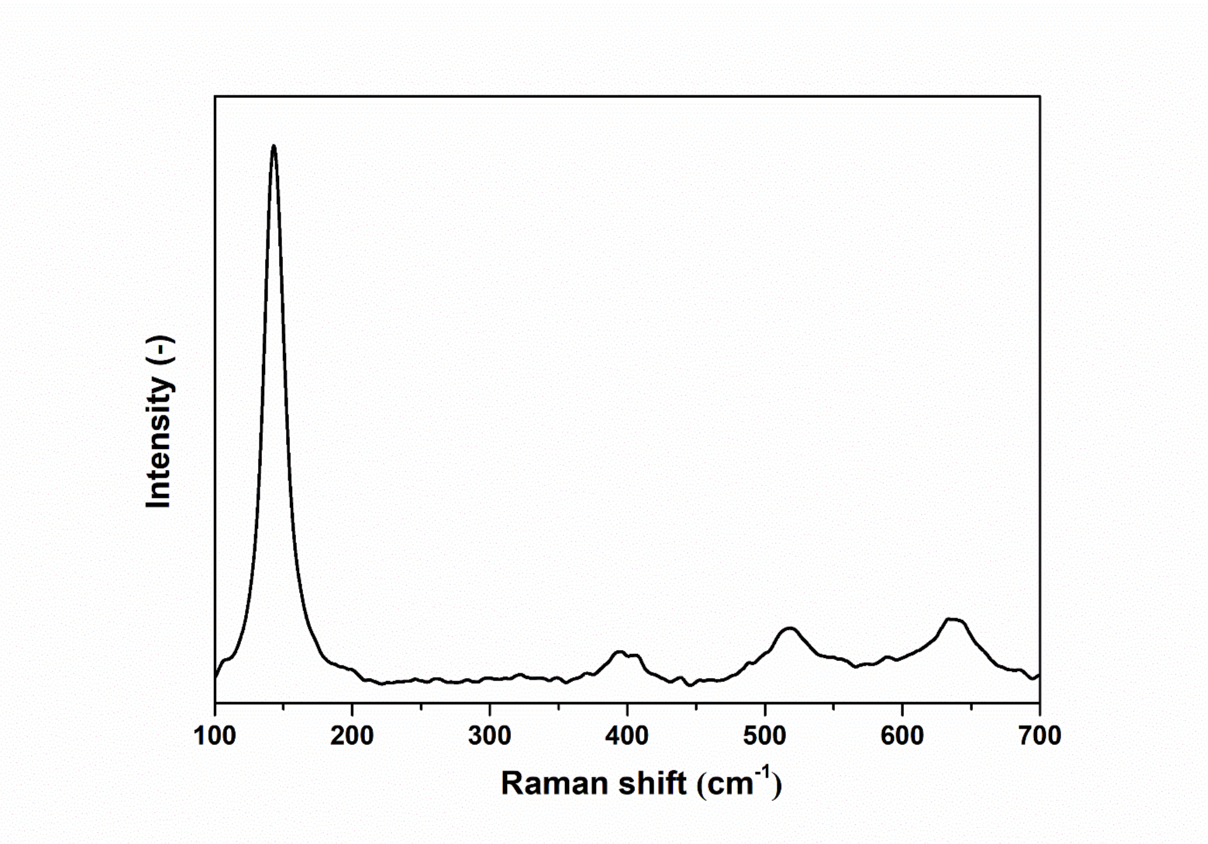

Fig. S5 Raman spectrum of TiO_2 thin film

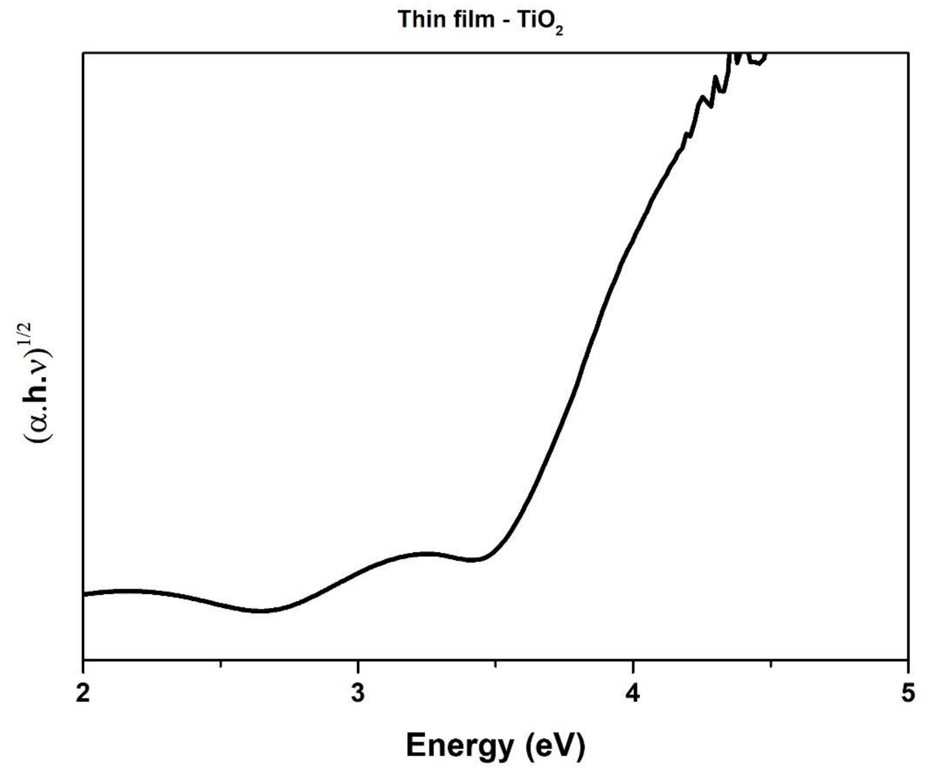

Fig. S6 UV-Vis spectrum of TiO_2 thin film

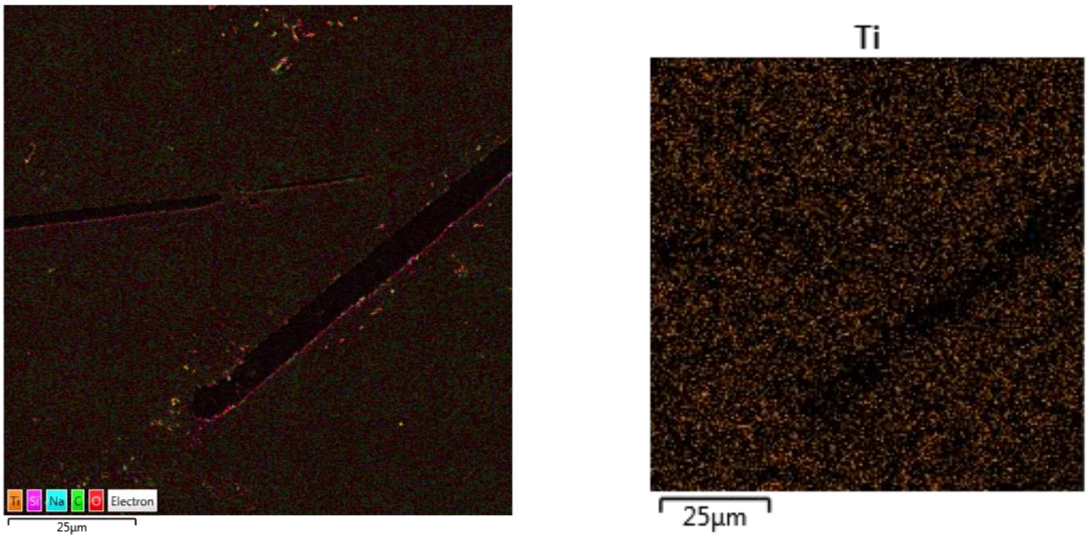

A B

Fig. S7 SEM EDX - Glass with a groove in a thin layer of TiO_2 film (A) and distribution of Ti (B)

Table S2 SEM-EDX – composition of TiO_2 thin film and borosilicate glass

Spectrum Label	TiO_2 thin film (SEM HV 5kV)	Borosilicate glass (SEM HV 5kV)	Borosilicate glass (SEM HV 20 kV)
		At. %	
B		10.07	
O	65.04	63.75	67.41
Na	0.88	0.06	1.09
Al	0.37	0.81	1.01
Si	10.87	25.3	30.18
K			0.3
Ti	22.83		

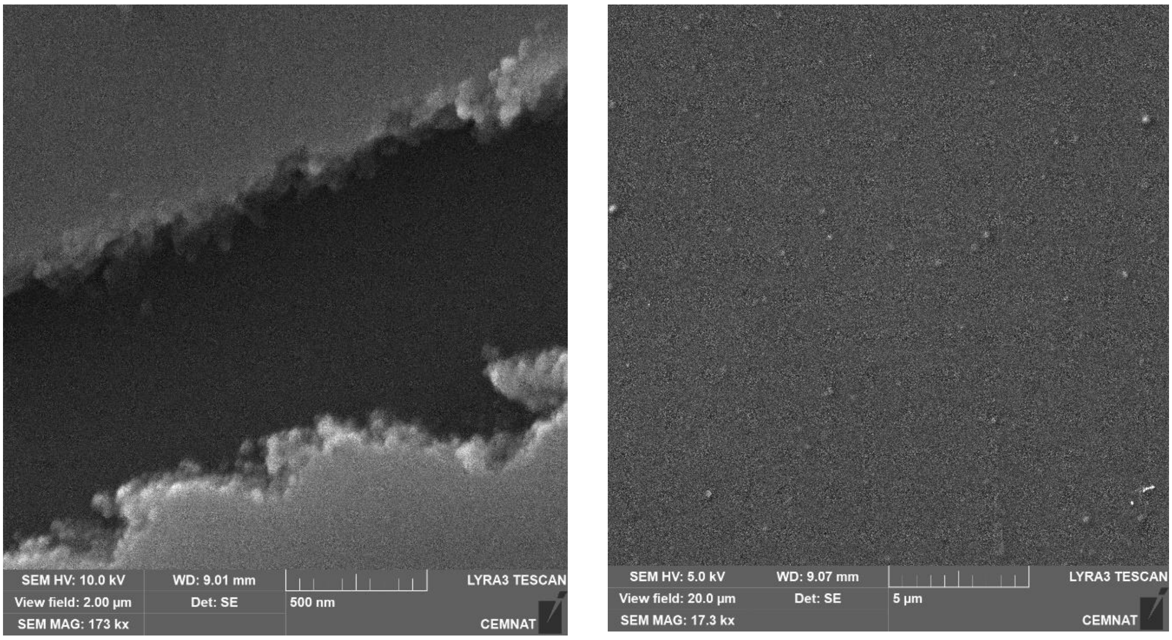

A **B**

Fig. S8 SEM - the groove in a thin layer of TiO_2 film (A) and pure TiO_2 thin film on borosilicate glass
